# Temporal variation and photochemical efficiency of species in Symbiodinaceae associated with coral *Leptoria phrygia* (Scleractinia; Merulinidae) exposed to contrasting temperature regimes

**DOI:** 10.1371/journal.pone.0218801

**Published:** 2019-06-28

**Authors:** Rodrigo Carballo-Bolaños, Vianney Denis, Ya-Yi Huang, Shashank Keshavmurthy, Chaolun Allen Chen

**Affiliations:** 1 Biodiversity Program, Taiwan International Graduate Program, Academia Sinica and National Taiwan Normal University, Taipei, Taiwan; 2 Biodiversity Research Center, Academia Sinica, Taipei, Taiwan; 3 Department of Life Science, National Taiwan Normal University, Taipei, Taiwan; 4 Institute of Oceanography, National Taiwan University, Taipei, Taiwan; 5 Department of Life Science, Tunghai University, Taichung, Taiwan; University of Connecticut, UNITED STATES

## Abstract

The Symbiodinaceae are paradoxical in that they play a fundamental role in the success of scleractinian corals, but also in their dismissal when under stress. In the past decades, the discovery of the endosymbiont’s genetic and functional diversity has led people to hope that some coral species can survive bleaching events by associating with a stress-resistant symbiont that can become dominant when seawater temperatures increase. The variety of individual responses encouraged us to scrutinize each species individually to gauge its resilience to future changes. Here, we analyse the temporal variation in the Symbiodinaceae community associated with *Leptoria phrygia*, a common scleractinian coral from the Indo-Pacific. Coral colonies were sampled from two distant reef sites located in southern Taiwan that differ in temperature regimes, exemplifying a ‘variable site’ (VS) and a ‘steady site’ (SS). We investigated changes in the relative abundance of the dominant symbiont and its physiology every 3–4 months from 2016–2017. At VS, 11 of the 12 colonies were dominated by the stress-resistant *Durusdinium* spp. (>90% dominance) and only one colony exhibited co-dominance between *Durusdinium* spp. and *Cladocopium* spp. Every colony displayed high photochemical efficiency across all sampling periods, while showing temporal differences in symbiont density and chlorophyll *a* concentration. At SS, seven colonies out of 13 were dominated by *Cladocopium* spp., five presented co-dominance between *Durusdinium* spp./*Cladocopium* spp. and only one was dominated by *Durusdinium* spp. Colonies showed temporal differences in photochemical efficiency and chlorophyll *a* concentration during the study period. Our results suggest that VS colonies responded physiologically better to high temperature variability by associating with *Durusdinium* spp., while in SS there is still inter-colonial variability, a feature that might be advantageous for coping with different environmental changes.

## Introduction

The success of coral reefs in tropical oligotrophic waters is often attributed to the symbiotic relationship between scleractinian corals and dinoflagellates algae. The Symbiodiniaceae are intracellular photosynthetic organisms that supply up to 95% of the coral host’s energy requirements in optimum conditions [1]. However, this subtle relationship is commonly disrupted under stressful environmental conditions, such as abnormally high seawater temperatures, in what is known as coral bleaching. Due to climate change and anthropogenic increases in seawater surface temperatures, bleaching events are becoming more frequent and severe [2]. Many coral reefs worldwide are now recurrently affected by mass bleaching events and mortality and, consequently, may become scarce within the next 20–30 years [3]. One well-known asset that some coral species have to survive bleaching events is their ability to associate with a functionally diverse community of symbionts, and to adjust their relative abundances to favour those better fitted to endure temperature variations [4–6].

The family Symbiodiniaceae is highly diverse and corals typically associate with members from the genera *Symbiodinium* (formerly Clade A), *Breviolum* (formerly Clade B), *Cladocopium* (formerly Clade C), and *Durusdinium* (formerly Clade D) [7]. *Durusdinium spp*. are extremophile endosymbionts that have been found in stressed habitats, such as those with high temperature [8–11], high turbidity [8] or located in high latitudinal marginal reefs [12–14]. In order to survive, some corals are able to increase the relative abundance of *Durusdinium trenchii* during and after bleaching events [15]. Some examples of this mechanism are found in species such as *Acropora millepora* in Australia [16]; *Orbicella annularis*, *Siderastrea siderea*, *Agaricia spp*., and *Montrastraea cavernosa* in Barbados [17]; and *Isopora palifera* in Taiwan [10]. It is a species- and location-specific mechanism that changes with environmental conditions [15, 18, 19]. Some examples include *Pocillopora damicornis*, *Seriatopora hystrix*, *Stylophora pistillata*, *Favites abdita*, *Goniastrea favulus*, *A*. *millepora*, *I*. *palifera* in Australia [18] and *O*. *faveolata* and *O*. *annularis* in the Florida Keys and the Bahamas [19], which did not change their symbiotic composition during and after thermal stress. Because the systematics of the Symbiodinaceae family is still a work in progress, there are many dinoflagellates that have not yet been identified to the species level. Therefore, the former sub-clade or type is used after the genera. Within the *Cladocopium* genus there are some species that have been recognized to be thermally resistant, such as *Cladocopium* C15. In Australia, *Porites lutea* colonies revealed that, when experimentally heated, corals hosting *Cladocopium* C15 maintained higher maximum photochemical efficiency (*Fv/Fm*) than those hosting *Cladocopium* C3 [20]. Similar results were found in *Porites lobata* from Hawaii; colonies that were experimentally bleached could maintain gross photosynthetic rates similar to control colonies when hosting *Cladocopium* C15. Moreover, this thermally tolerant symbiont helped the coral recover faster from bleaching by contributing 96% of the host’s daily metabolic demand, even when chlorophyll *a* levels were significantly lower than in the control [21].

The capacity to associate with multiple Symbiodinaceae genera is considered a widespread phenomenon [22, 23]. Some coral species can maintain a stable association with their dominant symbiont across their lifetime [24], including during and after stress events [18, 19]. Alternatively, some species are capable of shifting the relative abundance of their dominant partner to background symbionts when exposed to stress [5, 6, 25]. Those background symbionts are usually considered to initially represent <10% of the overall Symbiodinaceae community within one colony [26, 27]. A third scenario, documented for a very few number of species is to have a different symbiont dominating distinct areas within the coral colony (Table 1). In this study, we refer to multi-symbiont dominance on the colony scale, but each microhabitat within the colony can have its own dominant symbiont. For example, the dominant symbiont may be different between the top part of the colony and the lowest part. In the Caribbean, this has been documented in the *Orbicella* spp. complex (including *O*. *annularis*, *O*. *faveolata* and *O*. *franski*) since the 90’s (Table 1) [28–31]. These massive corals associate with multiple dominant symbionts in response to different light gradients, creating different microhabitats within the same coral colony. In *O*. *faveolata*—the top part of the colony, with high-irradiance—was dominated by *Symbiodinium* sp. and *Breviolum* sp., while the side or shaded parts of the colony were dominated by *Cladocopium* sp. [29, 31]. The same pattern was observed between colonies living at different depths: those living in shallow waters with high light intensity were dominated by *Symbiodinium* sp. and *Breviolum* sp., whereas *Cladocopium* sp. dominated those colonies living in deep waters [29]. In the Pacific, *Isopora palifera* colonies from southern Taiwan presented multi-symbiont dominance between *Cladocopium* C3 and *D*. *trenchii* (Table 1) [10, 32]. The relative abundance of both symbionts varied across the study period and *D*. *trenchii* became highly abundant in some colonies after the 1998 bleaching event [32]. The relative abundance of *D*. *trenchii* decreased afterwards and *Cladocopium* C3 became dominant almost 10 years after the bleaching event [10]. These differences in the dominant symbiont between Symbiodiniaceae genera explain why certain colonies, or parts of the colony, bleach and some do not during natural bleaching events [29]; they also explain how Symbiodiniaceae can cope with and recover from a bleaching event by shuffling their relative abundance [10]. These species are able to cope with environmental fluctuations, presenting a long-term ecological and evolutionary coral-Symbiodinaceae specialization strategy [29, 31].

**Table 1:** Studies reporting co-dominance of different symbiont genera within a single colony.

Host species	Symbiont genera/species	Study site	Genetic method for ID	Ref.
*Orbicella annularis*	*Symbiodinium*, *Breviolum*, *Cladocopium*	San Blas Archipelago, Panama	srRNA -RFLP	[28, 29]
*O*. *faveolata*	*Symbiodinium*, *Breviolum*, *Cladocopium*			
*O*. *annularis*	*Symbiodinium*, *Breviolum*, *Cladocopium*, *Durusdinium*	San Blas Archipelago, Panama	srRNA -RFLP	[30]
*O*. *faveolata*	*Symbiodinium*, *Breviolum*, *Cladocopium*, *Durusdinium*			
*O*. *franski*	*Symbiodinium*, *Breviolum*, *Cladocopium*, *Durusdinium*			
*Acropora tenuis*	*Cladocopium*, *Durusdinium*	Great Barrier Reef, Australia	rDNA-ITS1, SSCP	[33]
*A*. *valida*	*Cladocopium*, *Durusdinium*			
*A*. *valida*	*Cladocopium*, *Durusdinium*	Great Barrier Reef, Australia	rDNA-ITS1, SSCP	[34]
*Isopora palifera*	*Cladocopium*, *Durusdinium*	Kenting National Park, Taiwan	lsrRNA -RFLP	[32]
*O*. *faveolata*	*B*. *minutum*, *Cladocopium* C12	Lee Stoking Islands, Bahamas	ITS2-DGGE	[35]
*O*. *annularis*	*B*. *minutum*, *Brevolium* B10, *Cladocopium* C3, *D*. *trenchii*	Upper Florida Keys, USA		
*O*. *franski*	*B*. *minutum*, *Cladocopium* C12	Lee Stoking Islands, Bahamas		
*O*. *franski*	*B*. *minutum*, *Brevolium* B10, *Cladocopium* C3, *D*. *trenchii*	Upper Florida Keys, USA		
*Siderastrea siderea*	*Brevolium* B5a, *Cladocopium* C3	Upper Florida Keys, USA		
*O*. *annularis*	*B*. *minutum*, C7, *Cladocopium* C3	Carrie Bow Cay, Belize	ITS2-DGGE	[36]
*O*. *faveolata*	*Symbiodinium* A3, *B*. *minutum*, *Brevolium* B17, *Cladocopium* C7 and *D*. *trenchii*			
*Stephanocoenia intersepta*	*Symbiodinium* A3b, *Symbiodinium* A3, *Cladocopium* C3, *Cladocopium* C16, *Cladocopium* C54			
*A*. *valida*	*C*. *goreaui*, *Cladocopium* C2 and *Symbiodinium*	Great Barrier Reef, Australia	rDNA-ITS1, SSCP	[37]
*I*. *palifera*	*Cladocopium* C3, *D*. *trenchii*	Kenting National Park, Taiwan	ITS2-DGGE	[10]
*O*. *faveolata*	*Symbiodinium* A3, *Brevolium* B17, *Cladocopium* C7, *D*. *trenchii*	Puerto Morelos, Mexico	ITS2-DGGE	[38]
*O*. *faveolata*	*B*. *minutum*, *Cladocopium* C7a, *D*. *trenchii*	Exuma Cay, Bahamas	ITS2-DGGE	[31]
*O*. *faveolata*	*Symbiodinium* A3, *B*. *minutum*, *Brevolium* B17, *Cladocopium* C7	Carrie Bow Cay, Belize		
*O*. *faveolata*	*Symbiodinium* A3, *B*. *minutum*, *Brevolium* B17, *Cladocopium* C7, *D*. *trenchii*	Puerto Morelos, Mexico		
*O*. *faveolata*	*B*. *minutum*, *Cladocopium* C3	Upper Florida Keys, USA		

Studies in light grey are from the Indo-West Pacific Ocean.

In the present study, we describe the temporal dynamics of *Cladocopium* spp. and *Durusdinium* spp. associated with the sub-massive brain coral *Leptoria phrygia* (Ellis and Solander, 1786) in Kenting National Park, southern Taiwan. We monitored symbiont community changes and performance in coral colonies from two shallow reef flats exposed to contrasting seawater temperature regimes. The average shallow water temperatures at the ‘Outlet’ reef flat can be up to 2–3 °C higher than other coral reef sites during the summer [39, 40]. Furthermore, a tidal upwelling [41, 42] causes daily temperature fluctuation that can reach 6–8 °C; we considered this our “variable site” (VS). Temperatures at the ‘Wanlitong’ reef flat are steadier (daily seawater temperature fluctuations <3 °C); we considered this our “stable site” (SS). Our specific objectives were to: (1) investigate whether the relative abundance of endosymbionts changes are in line with temporal changes in local environments from both sites, and (2) characterise the dynamics of the endosymbionts’ physiology (photochemical efficiency, symbiont density, and chlorophyll *a* concentration) caused by these temporal changes.

## Materials and methods

### Study sites and temperature data

This study was conducted with proper permissions and permits (Field permit number: 1040008112) issued by the Kenting National Park authority.

**Fig 1:**
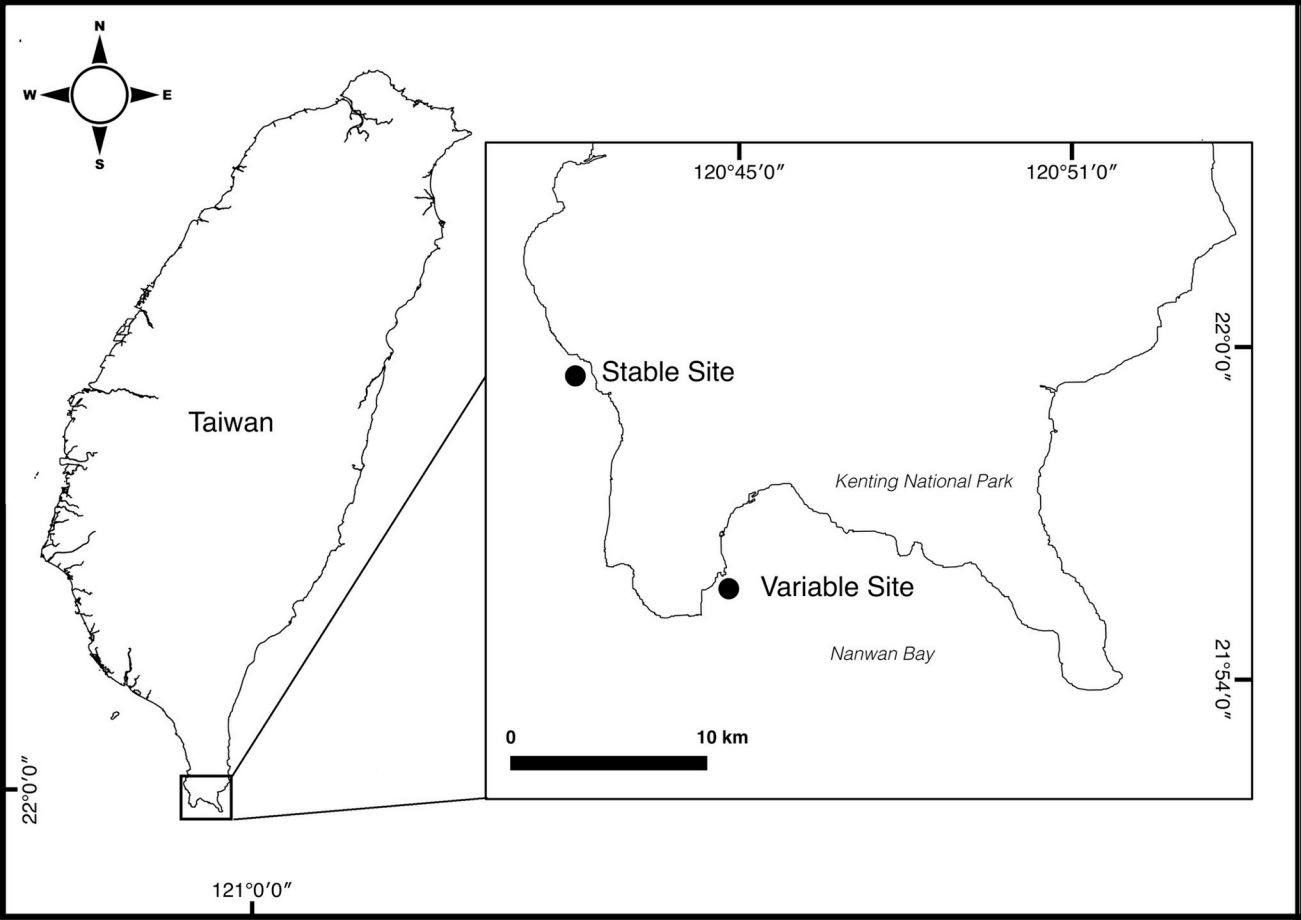
Study sites in Nanway Bay, southern Taiwan. Both sites are located within Kenting National Park: Variable Site (VS) in the power plant outlet (21°55'53.7"N 120°44'42.7"E) and Stable Site (SS) in Wanlitong (21°59'43.9"N 120°42'23.2"E).

Both Variable Site (VS) and Stable Site (SS) are located within Kenting National Park (KNP) in southern Taiwan (Fig 1). They are less than 20 km apart but have very different seawater temperature regimes. VS is located next to a nuclear power plant outlet in Nanwan Bay (21° 55' 53.7" N—120° 44' 42.7" E), where it is influenced not only by the hot-water effluent from the power plant throughout the year, but also by a spring tide upwelling which reduces the daily seawater temperature [40, 42]. SS is located outside Nanwan Bay (21° 59' 43.9" N—120° 42' 23.2" E) and is protected from the effects of the upwelling and the thermal pollution from the nuclear plant [11]. *Leptoria phrygia* is a common species at both sites, found in shallow waters and associated with *Durusdinium trenchii* in VS and *Cladocopium* C1 in SS [11]. Similarly, other species such as *Isopora palifera* and *Platygyra verweyi* living in VS were dominated by *D*. *trenchii* and associated with a mixture of *D*. *trenchii* and *Cladocopium* spp. or only with *Cladocopium* spp. at other sites in KNP [10, 11, 43, 44].

Temperature loggers (Onset HOBO 64K Pendant data loggers, accuracy ±0.5 °C, resolution 0.14 °C, USA) were deployed at a depth of 3 m at each site and temperatures were recorded at 1 hr intervals from June 2016 to June 2017. The mean monthly temperature, mean monthly maximum and minimum temperatures and mean monthly temperature variability (defined as the mean monthly maximum temperature minus the mean monthly minimum temperature) were calculated at each site for comparison.

### Sample collection, photochemical efficiency and preservation

Twelve and 13 large colonies were randomly selected at VS and SS, respectively, and tagged with a minimum distance of 7–10 m among them to avoid picking up clones. Coral colonies were sampled at an interval of 3–4 months (in August, December 2016 and March 2017) to characterize seasonal differences typifying the tropical monsoon climate of Kenting. Five nubbins were collected from each colony using a pneumatic drill connected to a regulator on a scuba diving tank and equipped with a core bit (∅ 2.8 cm). Nubbins were sampled at distances of 10–20 cm apart longitudinally from the top to the bottom of the colony (top part of the colony, top middle part, middle part, bottom middle part and bottom part). All collected nubbins were transported in coolers filled with seawater to the wet laboratory of the nearby National Museum of Marine Biology and Aquarium (NMMBA) and placed in tanks under flowing seawater to emulate field conditions. Dark-adapted maximum photochemical efficiency (*Fv/Fm*) of PSII was quantified using a diving-PAM (Heinz Walz GmbH, Germany; settings: Saturating intensity = 8, Saturating width = 0.8, Gain = 4, Damping = 2), keeping a fixed distance between the fibre and the sample by attaching a 1 cm probe extension. Dark-adapted (*Fv/Fm*) measurements were done approximately two hours after sunset, due to logistical constraints. A test was performed to see if (*Fv/Fm*) values after two hours were different from those after only 30 minutes for *L*. *phrygia*; results showed similar values. Nubbins were then immediately snap-frozen in liquid nitrogen and stored at -80 °C for transportation and further processing.

### Laboratory analyses

Each coral sample was defrosted, inspected for contaminants, and a small subsample of coral tissue (~6 mm) was cut and stored in an Eppendorf tube with 95% Ethanol for molecular analysis. Remaining coral tissue was then air-brushed [45] from the skeleton using approximately 15 ml of filtered seawater (0.2 μm). Resulting slurry (*Vi*) was homogenized using a Homogenizer Stirrer (WiseStir HS-30E, Germany).

### Surface area

The surface area of the remaining coral skeleton was measured using a 3D scanner (HP David SLS-2 3D structured light scanner Pro S3, resolution/precision up to 0.1% of scan size down to 0.06 mm, max mesh density: 1,200,000 vertices per scan; USA) equipped with a 360 ° turntable and area calculations were done using the open source software Meshlab [46]; data were presented in cm^2^.

### Symbiont density

Symbiont density was determined from a 10% aliquot of *Vi* by counting cells with 6–8 replicates per sample using a Neubauer hemocytometer (Assistant, Germany) under a light microscope (BX40 Olympus, Japan). This cell count was then normalized to the surface area of the nubbin, and presented as number of symbiont cells per cm^2^.

### Chlorophyll *a* concentration

Another 10% aliquot of *Vi* was centrifuged at high speed (5 min, 14000 xg). The supernatant was removed; 1 ml of 90% acetone was added to the pellet and incubated in total darkness at -20 °C overnight for photosynthetic pigment extraction [47]. After centrifugation for 1 min at 10000 xg, supernatant absorbance was read at 630, 647 and 664 nm wavelengths from triplicate aliquots of 200 μl each, using a spectrometer (SpectroStar nano absorbance reader BMG-LabTech, Germany) with three wells containing 200 μl of 90% acetone as a blank. The chlorophyll *a* concentration was then calculated using the equations from Jeffrey and Humphrey [48]: Chl*a* = 11.85 A_664_−1.54 A_647_−0.08 A_630_. Chlorophyll *a* concentration was then normalized per cm^2^ of the nubbin’s surface area and the symbiont density and presented as pg of chlorophyll *a* concentration per symbiont cell per cm^2^.

### Molecular analysis

#### DNA extraction

Genomic DNA was extracted using a modified high salt method [49]. Briefly, for tissue incubation, 30 mg of coral tissue (~ 3–4 polyps) was cut and incubated overnight at 55–60 °C using 200 μl lysis buffer (1M Tris-Boric 25 ml, 0.5M EDTA pH8 10 ml, 20% SDS 10 ml, 5M NaCl 2 ml, ddH_2_O 53 ml) and 10 μl proteinase E (10 mg/ml). For DNA precipitation, 210 μl 7M-NaCl was added, vortexed for 30 seconds at maximum speed and centrifuged for 30 minutes at 10000 xg. The supernatant was transferred to a new tube and 420 μl of 100% isopropanol were added and gently mixed for five minutes. Samples were incubated at -20 °C for at least two hours. For DNA purification, samples were centrifuged (30 minutes at 16000 xg) and rinsed with 150 μl of 70% ethanol stored at -20 °C and centrifuged again (5 minutes at 16000 xg); this rinsing step was repeated three times. Ethanol was removed from the tubes, which were dried under the hood for 1–2 hours. The DNA was eluted with 150 μl of preheated (65 °C) 1X TE buffer. The total concentration of genomic DNA was determined using a NanoDrop 2000 (Thermal Scientific, USA).

#### Quantitative PCR (qPCR)

Relative abundances of *Cladocopium* sp. and *Durusdinium* sp. were measured by amplifying the ITS1 region using the LightCycler 480 Instrument II (Roche, Switzerland) with a modified protocol from Mieog *et al*. [26]. The following primers were used: nuclear ITS1 universal forward primer (UF, 5’-AAGGAGAAGTCGTAACAAGGTTTCC-3’), nuclear ITS1 C-specific reverse primer (CR, 5’-AAGCATCCCTCACAGCCAAA-3’), and ITS1 D-specific reverse primer (DR, 5’-CACCGTAGTGGTTCACGTGTAATAG-3’) [34]. Each 10 μl qPCR reaction consisted of 5 μl of 1x SYBR Fast Master Mix, 0.5 μl of UF primer (2 nM/μl), 0.5 μl of CR or DR primer (2 nM/μl), 1.5 μl of ddH_2_O and 2.5 μl of DNA templates (equal to 1 ng of genomic DNA). The two-step qPCR reactions were set at 95 °C for 15 seconds and 60 °C for one minute. 40 cycles were performed in total. Melting curves were generated to start at 60 °C with an increase of 0.11 °C/s until it reached 95 °C, then followed by a cooling step to 40 °C for 30 seconds. The ratio of *Durusdinium* (D) to *Cladocopium* (C) was calculated using the formula: ratio = D/(C+D) from triplicate measures per sample. A correction was done to account for the differences in copy numbers between *Cladocopium* and *Durusdinium* genera following Mieog *et al*. [26].

#### Symbiont identification—(DGGE)

Denaturing gradient gel electrophoresis (DGGE) was used for symbiont identification. The ribosomal internal transcribed spacer 2 (ITS2) region was amplified with the primers ITSintfor2: 5’-GAATTGCAGAACTCCGTG-3’, and ITS2clamp: 5’-CGCCCGCCGCGCCCCGCGCCCGTCCCGCGGGATCCAT-ATGCTTAAGTTCAGCGGGT-3’ and touch-down PCR protocol [50]. Each PCR product was loaded onto an acrylamide denaturing gradient gel (45–80%) and then electrophoresed at a voltage of 115 for 15 hours (CBS Scientific system, USA). Gels were stained with SYBR gold (Invitrogen, USA) for 30 minutes and photographed for further analysis. The most significant band of each PCR product was cut from the gel and eluted in 100 μl of distilled water for a few hours. Re-amplification was performed with 1 μl of the sample following a protocol similar to touch-down PCR-DGGE, but using a reverse ITS2 primer without the clamp [50], and the product was sent for sequencing. The resulting sequences were trimmed, cleaned and blasted against the database of the National Center for Biotechnology Information (NCBI) to determine Symbiodiniaceae genus.

### Statistical analysis

Mean monthly temperature, mean maximum/minimum temperature and mean monthly temperature variability (defined as the mean monthly maximum minus the mean monthly minimum temperature) were calculated for each site and the differences between VS and SS during the study period were tested using the non-parametric Mann-Whitney-Wilcoxon test after confirming that data from most months were not normally distributed. Data from all measured physiological parameters were checked for normality and homogeneity of variances (Q-Q plot and Fligner test) and all were normally distributed and presented homoscedasticity. A linear mixed model was used to test the effect of sampling times as the fixed effect (with three levels) at each site, using colony as the random effect. Similarly, to compare between sites at each sampling time, a linear mixed model was used with site as the fixed effect (with two levels) and using colony as the random effect. P-values were obtained by likelihood ratio test of the full model against the model without the effect in question. This was performed for each of the physiological parameters measured. All samples at both sites were combined and the non-parametric Spearman correlation was used to test for monotonic correlations between each temperature measurement independently (mean monthly temperature, mean monthly maximum temperature, mean monthly minimum temperature and mean monthly temperature variability) and *Durusdinium* spp. association. All statistical analyses were performed in R (version 3.4.1) [51], using the ‘lme4’ package for the linear mixed model analysis [52]. All data were presented as mean ± standard deviation (S.D.).

## Results

### Seawater temperature differences between sites

**Fig 2:**
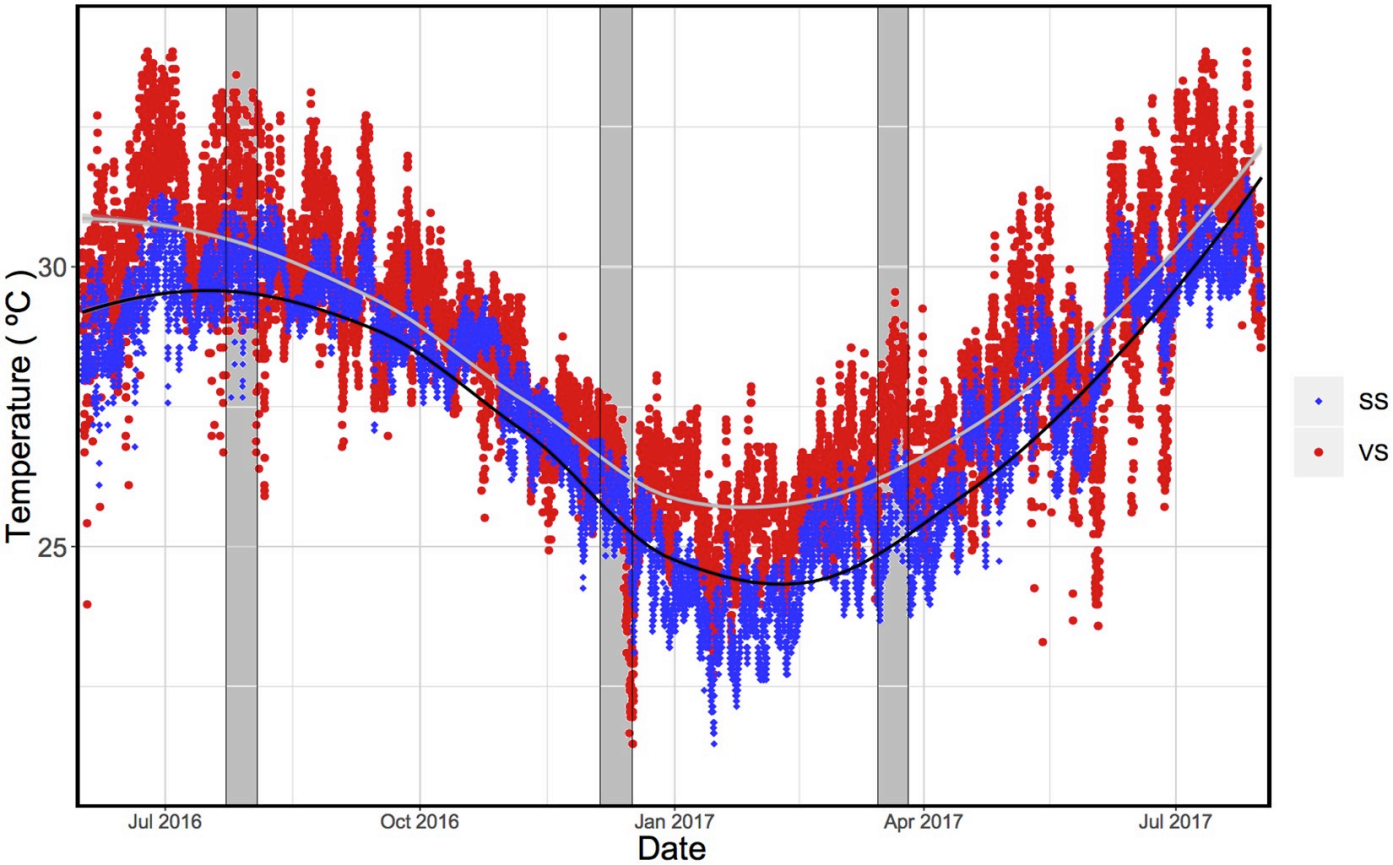
Seawater daily temperatures recorded for June 2016 to July 2017 at both sites. SS = Stable Site, VS = Variable Site. Each dot represents hourly measurements. The black line indicates the mean daily temperature in SS and the grey line represents the mean daily temperature in VS. Vertical grey bands represent each sampling time (August, December 2016 and March 2017).

Both sites displayed different seawater temperature regimes (Fig 2) from June 2016 to June 2017. Mean monthly temperature in VS ranged from 25.4 ± 0.9 °C to 31.0 ± 1.1 °C while in SS it ranged from 23.7 ± 0.7 °C to 29.7 ± 0.6 °C, making it significantly higher in VS than in SS (Mann Whitney test, p<0.05). June 2017 was the only month in which there was no significant difference in mean monthly temperatures between sites (Mann Whitney test, p = 0.58). During the summer of 2016, July exhibited the maximum mean temperature of the year in VS (32.3 ± 0.9 °C), which was significantly different to that in SS (30.4 ± 0.6 °C). In the winter of 2017, January showed the minimum mean temperature of the year in VS (24.6 ± 0.9 °C), which was significantly different from that in SS (23.1 ± 0.7 °C; Table in S1 Table). The mean monthly temperature variability (mean monthly maximum temperature minus the mean monthly minimum temperature) was also significantly different between sites. Mean monthly temperature variability was significantly higher in VS (1.5 ± 0.5 °C to 3.2 ± 1.1 °C) than in SS (0.7 ± 0.4 °C to 1.6 ± 0.8 °C) during the study time (Mann Whitney test, p<0.05) (Fig 2, S1 Table).

### Symbiodinaceae association

**Fig 3:**
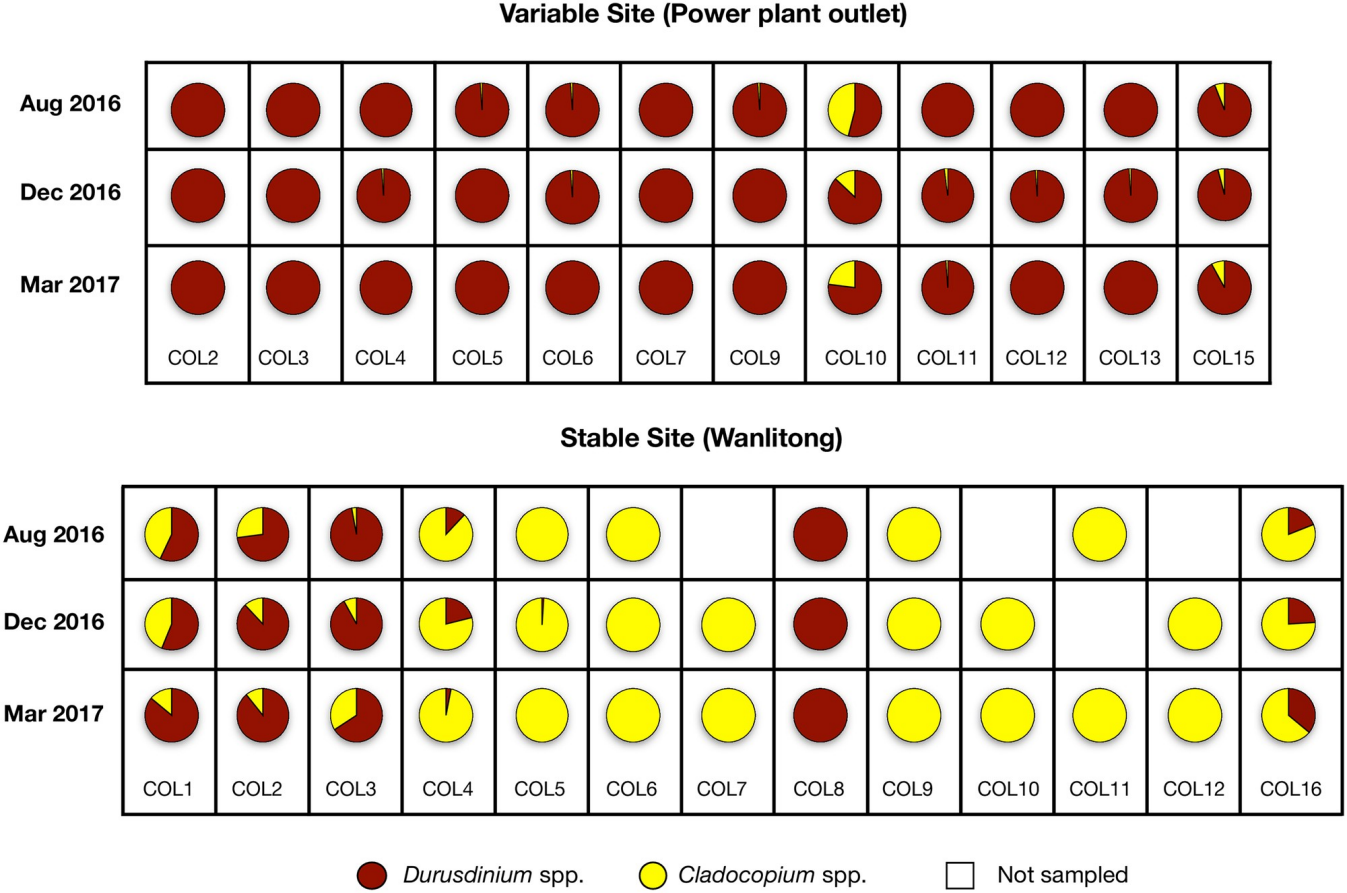
Pie charts showing symbiont associations between *Cladocopium* spp. and/or *Durusdinium* spp. Each pie chart represents the mean values per colony during three sampling times in August 2016 (Aug 2016), December 2016 (Dec 2016) and March 2017 (Mar 2017); variable site n = 12 colonies (top) and stable site n = 13 colonies (bottom).

Among the 12 colonies sampled in VS, *Durusdinium* spp. dominated in 11 (>90%), and none showed temporal variation across the entire study period. One colony exhibited co-dominance between *Cladocopium* spp. and *Durusdinium* spp. (COL10, Table 2) and presented variation: *Durusdinium* spp. dominated the Symbiodinaceae community in August 2016 (54%), increased in December 2016 (87%) and decreased in March 2017 (77%, Fig 3). Of the 13 colonies at SS, seven were dominated by *Cladocopium* spp. (>90%), five presented co-dominance between genera in *Cladocopium* and *Durusdinium*, and only one was *Durusdinium* spp.-dominant (>90%; Fig 3). Similar to what was observed in VS, there was no variation in those colonies that were either *Cladocopium* spp.- or *Durusdinium* spp.-dominant during the entire study period. Only those five *Cladocopium* spp./*Durusdinium* spp. co-dominated colonies presented temporal variability (Fig 3). (For more detailed information see S2 Table).

**Table 2:** Dominant symbiont identified with DGGE and subsequent sequencing at both sites.

	VS	SS
COL1	-	*D*. *glynnii* / *Cladocopium* sp.
COL2	*D*. *glynnii*	*D*. *glynnii* / *Cladocopium* sp.
COL3	*D*. *glynnii*	*D*. *glynnii* / *Cladocopium* C3w
COL4	*D*. *glynnii*	*D*. *glynnii* / *Cladocopium* C3w
COL5	*D*. *glynnii*	*Cladocopium* C3w
COL6	*D*. *glynnii*	*Cladocopium* C21a
COL7	*D*. *glynnii*	*Cladocopium* C3w
COL8	-	*D*. *glynnii*
COL9	*D*. *glynnii*	*Cladocopium* C3w
COL10	*D*. *glynnii* / *Cladocopium* C21a	*Cladocopium* C21a
COL11	*D*. *glynnii*	*Cladocopium* C21a
COL12	*D*. *glynnii*	*Cladocopium* C3w
COL13	*D*. *glynnii*	-
COL15	*D*. *glynnii* / *Cladocopium* C3w	-
COL16	-	*D*. *glynnii* / *Cladocopium* C3w

VS = Variable Site, SS = Stable Site.

**Fig 4:**
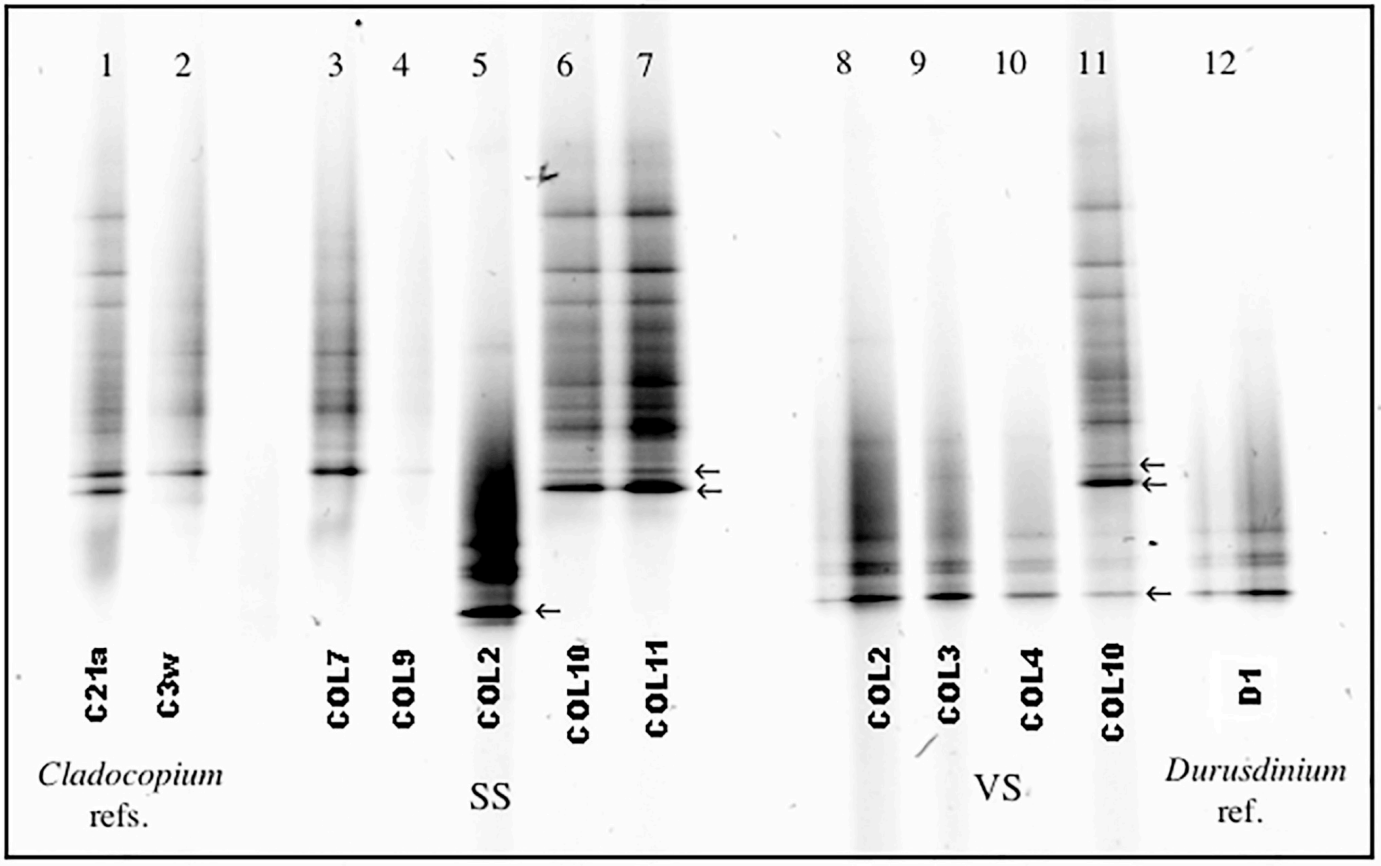
Denaturing gradient gel electrophoresis (DGGE) from selected colonies at each site. SS = Stable Site, VS = Variable Site. Arrows indicate the main bands excised for sequencing as examples. Lanes 1 and 2 represent *Cladocopium* C21a (with double bands) and *Cladocopium* C3w references, respectively. Lane 12 represents *D*. *glynnii* reference. Lanes 3–7 are colonies from SS dominated either by *Cladocopium* C3w (COL7 and COL9) or *Cladocopium* C21a (COL10 and COL11) or co-dominated by *Cladocopium* sp. and *D*. *glynnii* (COL2). Lanes 8–10 are colonies from VS dominated by *D*. *glynnii* (COL2, COL3 and COL4) and lane 11 is the colony with co-dominance between *Cladocopium* sp. and *D*. *glynnii*.

DGGE and subsequent sequencing of the major bands confirmed that *D*. *glynnii* (GenBank accession number MK127922) was the most dominant *Durusdinium* endosymbiont found at both sites. There was also inter-colonial variation in those *Cladocopium* spp.-dominated colonies in SS. *Cladocopium* C3w (GenBank accession number MK127920) and *Cladocopium* C21a (GenBank accession number MK127921) were the two most dominant endosymbionts found in different colonies (Table 2, Fig 4). This difference between *Cladocopium* spp. was identified in the DGGE band profiling (Fig 4) and later confirmed by sequencing. *Cladocopium* C3w showed only one dominant band in the DGGE profile (Fig 4) and was dominant in five colonies (COL4, COL5, COL7, COL9, and COL12; Table 2). *Cladocopium* C21a displayed two bands (heteroduplex) in the DGGE profile (Fig 4) and was dominant in three colonies (COL6, COL10 and COL11; Table 2).

### Correlation between *Durusdinium* spp. and temperature

**Fig 5:**
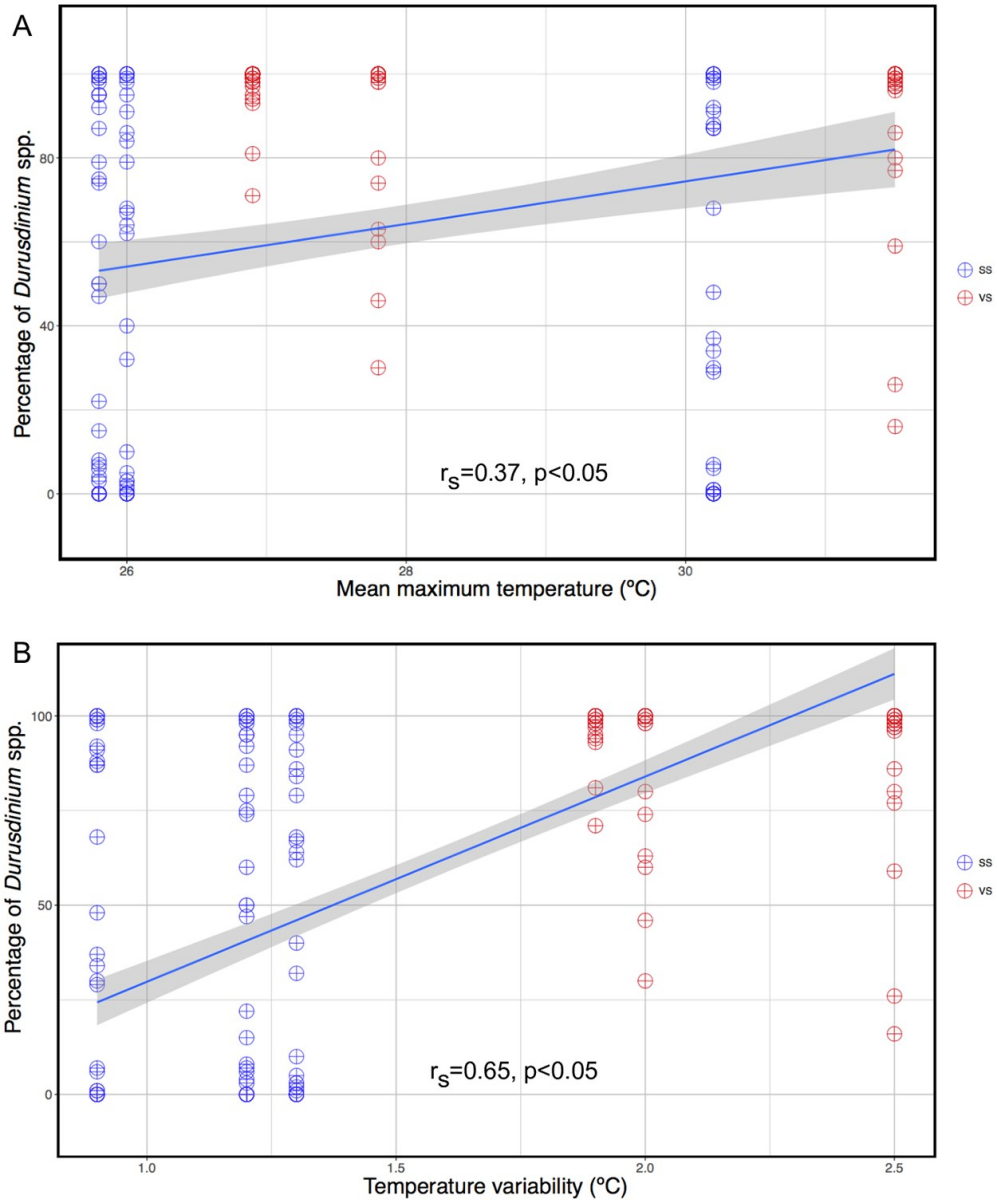
Correlation between mean maximum temperature and the percentage of *Durusdinium* spp. in A and between delta temperature (mean monthly maximum–mean monthly minimum) and the percentage of *Durusdinium* spp. in B. SS = Stable Site, VS = Variable Site; each dataset represents a sampling time. The shaded area represents 95% confidence interval.

We found significant positive monotonic correlation (r_s_ = 0.37, p<0.05) between the mean maximum temperature and the percentage of *Durusdinium* spp (Fig 5A), as well as between the temperature variability and the percentage of *Durusdinium* spp. (r_s_ = 0.65, p<0.05) during the study period (Fig 5B).

### Physiological parameters

**Fig 6:**
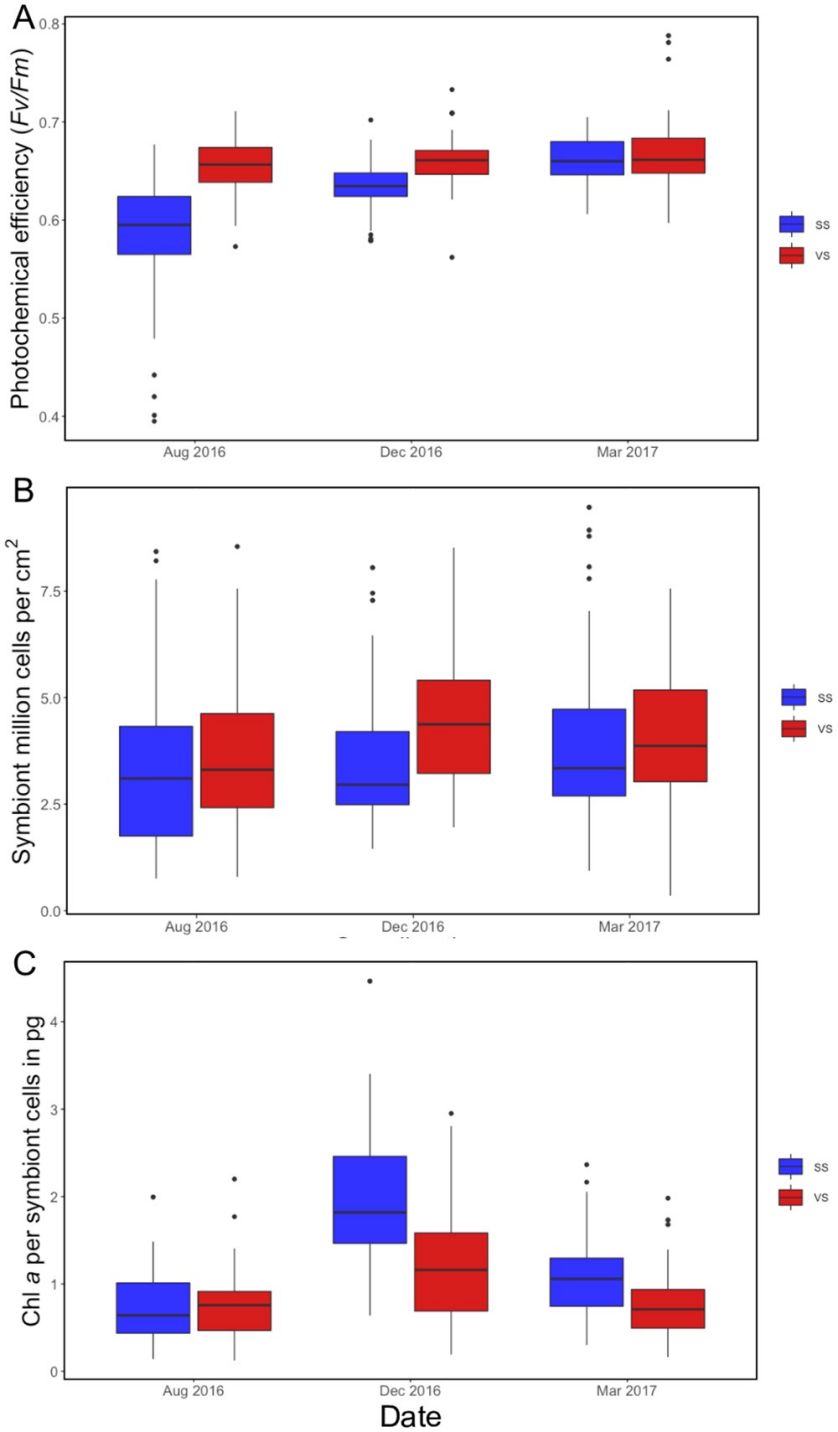
Physiological parameters measured photochemical efficiency (*Fv/Fm*) in A, symbiont density (million cells cm^-2^) in B and chlorophyll *a* concentration (pg per symbiont cells) in C. All measurements present the median values of all colonies together per sampling time: August 2016 (Aug 2016), December (Dec 2016) and March 2017 (Mar 2017); SS = Stable Site, VS = Variable Site.

In VS, there was no temporal variation and no significant difference in photochemical efficiency across all sampling times (χ^2^(2) = 4.90, p = 0.09) and coral colonies maintained similar mean *Fv/Fm* during all sampling months (August: 0.66 ± 0.03, December: 0.66 ± 0.03 and March: 0.67 ± 0.04; Fig 6A). In contrast, colonies in SS presented significant temporal variation (χ^2^(2) = 98.00, p<0.05), with a mean *Fv/Fm* of 0.58 ± 0.06 in August (2016), 0.63 ± 0.02 in December (2016) and 0.66 ± 0.02 in March (2017) (Fig 6A). Photochemical efficiency was statistically different between the two sites in August (χ^2^(1) = 53.01, p<0.05) and December 2016 (χ^2^(1) = 30.13, p<0.05) but not in March 2017 (χ^2^(1) = 2.08, p = 0.15).

There was temporal variation in symbiont density in VS. Mean values were significantly different between months (χ^2^(2) = 7.99, p<0.05; Fig 6B), increasing from 3.70 ± 1.66 x10^6^ cells cm^-2^ (August 2016) to 4.54 ± 1.68 x10^6^ cells cm^-2^ (December 2016), then decreasing again to 4.06 ± 1.53 x10^6^ cells cm^-2^ (March 2017). However, in SS there was no significant difference in density between sampling times (χ^2^(2) = 3.78, p = 0.15; Fig 6B). Symbiont density was only statistically different between sites in December 2016 (χ^2^(1) = 12.70, p<0.05).

Chlorophyll *a* concentration was significantly different between the two sites in December 2016 (χ^2^(1) = 31.74, p<0.05) and March 2017 (χ^2^(1) = 21.53, p<0.05), but showed no significant difference in August 2016 (χ^2^(1) = 0.03, p = 0.87). Within each site, chlorophyll *a* concentration was also significantly different across sampling times (χ^2^(2) = 33.90, p<0.05 for VS; χ^2^(2) = 116.2, p<0.05 in SS). Chlorophyll *a* concentration increased at both sites from August to December 2016 and decreased again in March 2017 (Fig 6C).

## Discussion

In this study we characterised Symbiodiniaceae communities of *Leptoria phrygia* from two sites with contrasting temperature regimes. This species has been reported to associate with *Cladocopium* and *Durusdinium* in American Samoa [9], and, in KNP, southern Taiwan, *L*. *phrygia* is also known to host both genera within the same colony [11]. Our study demonstrates that the stress-resistant *Durusdinium* spp. dominated almost all colonies in VS. In contrast, coral colonies in SS presented three different types of associations: 1) dominated by *Cladocopium* (54%), 2) co-dominated by *Cladocopium* and *Durusdinium* (39%) and 3) dominated by *Durusdinium* (7%). Nonetheless, in both VS and SS, those colonies dominated by a single genus—i.e., *Durusdinium* or *Cladocopium* only—continued to be dominated by that genus through time. Even the dominant species within those colonies remained the same, e.g., *Cladocopium* C3w or *Cladocopium* C21a in the *Cladocopium*-dominated colonies and *D*. *glynnii* in the *Durusdinium*-dominated colonies.

However, there was distinct temporal variation in colonies with multi-symbiont dominance (co-dominated by two genera), particularly those from SS.

*Durusdinium* can tolerate 1.0 to 1.5 °C higher than *Cladocopium* in *Acropora millepora* [5]. Our results showed that there is a significant positive correlation between the mean maximum temperature and the percentage of *Durusdinium* spp. a phenomenon also documented under natural conditions in previous studies [9, 53, 54]. We also found significantly higher correlation between high temperature variability and the percentage of *Durusdinium* spp. That might be the main reason why almost all colonies in VS are associated with the stress-resistant *Durusdinium* spp. Other studies also found that coral communities living at this thermally variable site were dominated by *D*. *trenchii* [10, 43, 44]. Another survey done at the same site reported that species from the genera *Acropora*, *Cyphastrea*, *Goniastrea*, *Isopora*, *Platygyra*, *Favites*, *Pocillopora*, *Acanthastrea*, and *Leptoria* associated with *D*. *trenchii* at 3 m deep in VS, while at 7 m deep the same species associated with *Cladocopium* spp. (C1, C3, C21a and C15) [11]. This difference in symbiont communities between depths implies that the hot water from the power plant outlet only affects the shallow waters in VS [11].

Our results indicate that, in addition to high temperature, large temperature variability also plays an important role in determining symbionts associations; this concurs with a study of four common taxa sampled from four different sites with a gradient of temperature variability in Kenya over 10 years [55]. The study showed that *Pavona* and *Pocillopora* were the genera that best survived bleaching events, including the global bleaching event of 1998; similar to our study, most colonies in these two genera lived in the sites with the highest temperature variability and were associated with *Durusdinium* sp. [55].

Those colonies in SS presenting intra-colonial variation, plus a *Durusdinium* spp.-dominated colony (46% of all colonies sampled), have the stress-resistant symbiont that provides higher resilience to bleaching events than other taxa. Those *Cladocopium*/*Durusdinium* co-dominated colonies presented temporal variability in the dominant symbionts, which offers a ground for corals to shuffle the relative abundances of their dominant symbiont [56]. This suggests that *L*. *phrygia*’s inter-colonial variation might be an ecological advantage, because coral species that host symbionts resistant to variable conditions will most likely survive climate warming events [6, 23, 24, 57].

This theory that inter-colonial variation provides ecological advantage has been experimentally simulated with two species in the Caribbean with multi-symbiont associations, *Montastraea cavernosa* and *O*. *faveolata* [6, 57]. Corals were stressed with high temperatures to induce bleaching. They recovered by increasing their abundance of the resistant symbiont *D*. *trenchii* and maintaining this association after recovery for three months at 29 °C [6, 57]. Researchers exposed *M*. *cavernosa* corals to a subsequent bleaching heat stress and determined that those *D*. *trenchii*-dominated corals were more resistant to bleaching and lost fewer symbionts than before [6].

*Durusdinium trenchii* has been found to increase bleaching thresholds by 1.0 to 1.5 °C under high temperature stress [5, 6, 58] by maintaining a high photochemical efficiency compared to other symbionts from the *Breviolum* and *Cladocopium* genera [25, 59]. Our study suggests that species living in VS are able to resist high summer temperatures and temperature variability by hosting *Durusdinium* spp. Colonies in VS did not display any signs of stress when their maximum photochemical efficiency was measured, even during the summer, when mean maximum temperatures reached 32.3 ± 0.9 °C and were significantly higher than in SS. All colonies in this highly variable thermal environment showed mean *Fv/Fm* higher than 0.66 ± 0.03 (see Fig 6A).

In the southern Great Barrier Reef, *A*. *valida*, a species dominated by multiple *Cladocopium* and *Symbiodinium* symbionts, did not present seasonal variation in photochemical efficiency [37]. But the lack of seasonal variation was explained by changes in the relative abundance of its symbionts, because most colonies increased their percentage of *Symbiodinium* sp. after a bleaching event in the summer. Therefore, they maintained high photochemical efficiency during the winter [37]. In contrast, SS colonies varied their photochemical efficiency through sampling times by increasing *Fv/Fm* during the winter. Similar results were found in the Caribbean in *Orbicella* spp. [60] and in the South China Sea in three *Acropora spp*., *Pavona decussata* and *Porites lutea* [61]. *Durusdinium* spp. not only are advantageous to corals under high temperature, they can also resist cold temperature stress [62].

*M*. *cavernosa* corals dominated by either *D*. *trenchii* or *Cladocopium* C3 were experimentally exposed to temperatures down to 15 °C and, when measuring the symbiont photochemical efficiency, both coral colonies showed photodamage. Those corals dominated by *D*. *trenchii* neither bleached nor died, while *Cladocopium* C3-dominated corals bleached, losing 94% of their symbionts [62]. Similarly, *Pocillopora* spp. corals associated with *D*. *glynnii* better survived a cold bleaching event in 2008 in the Gulf of California [63]. All colonies associated with *Cladocopium* C1b-c bleached during the cold event and 56% suffered partial or total mortality, whereas those colonies associated with *D*. *glynnii* neither bleached nor showed any mortality [63].

During our study period, there was no sign of bleaching from stress at either site, and corals in VS exhibited an increase in symbiont density during December 2016 following natural temporal fluctuations, which caused a significant difference between sites. In SS, there was high variability between colonies in 2016, therefore mean values of symbiont density in December were comparable to those in August.

Similar results as in VS were found for *A*. *muricata* in a long-term monitoring study (6 years) in Mauritius: symbiont densities were three times higher during the autumn and winter than the spring and summer, and the authors found that symbiont density positively correlated with nitrate concentrations [64]. In the Caribbean, a long-term monitoring study (4 years) found similar results: *A*. *cervicornis*, *A*. *palmata*, *O*. *annularis* and *O*. *faveolata* presented higher values during the winter [47]. A study in the Red Sea described this same seasonal variation in *P*. *verrucosa*, with higher densities during the winter [65].

This difference in symbiont density between seasons has been explained by dynamic photoinhibition, where carbon fixation decreased due to down-regulation of photosynthesis when there is high light, such as during the summer [47]. Additionally, chlorophyll *a* concentration values showed temporal fluctuations due to this dynamic photoinhibition related to high light and temperature during the summer [60, 66, 67]. Both sites in our study presented lower values during the summer and increased values in the winter.

In summary, understanding the physiological plasticity of a species such as *Leptoria phrygia* living in different environments allows us to establish different mechanisms that species could use to withstand future climate change. Here, we examined the multi-symbiont association in *L*. *phrygia* in a stable environment and compared it to the same species living at a site with high temperature variability. Our results suggest that those corals living in variable environments provide important information on coral resilience during environmental perturbations. Coral colonies in VS dominated by *Durusdinium* spp. had better physiological responses and were able to cope better with the high variability in seawater temperature. These results raise the question of whether the symbiont community of coral species such as *L*. *phrygia* may have been selected by the environmental conditions they live in. In addition, only those *Durusdinium*/*Cladocopium*-dominated colonies presented temporal variability. No matter the environmental conditions they lived in, those *Durusidinum* spp.- and *Cladocopium* spp.-dominated colonies maintained the same dominant symbiont at all times. These results also question the role of the coral host in deciding which Symbiodinaceae community to associate with. In order to answer these questions and to have a better understanding of how highly variable environments will respond to future climate change, we recommend further experimental work between both sites, such as transplantation experiments; we also recommend measuring the physiological parameters of all partners in the holobiont. It will also be interesting to investigate if species already dominated by *Durusdinium* spp. will be able to survive more recurrent bleaching events [2] or prolonged thermal stresses [44] in the future.

## Supporting information

S1 Table Seawater temperature information at both sites from June 2016 to June 2017. Months in which sampling was performed are in grey. Abbreviations: MMT° = Mean monthly temperature, MMMax T° = Mean monthly maximum temperature, MMMin T° = Mean monthly minimum temperature, ΔT° = maximum temperature-minimum temperature, VS = Variable Site, and SS = Stable Site.
(PDF)

S2 Table Percentage of *Durusdinium* spp. at both sites. (PDF)

S1 Dataset Raw data set with mean values from Figs 3 and 6. (XLSX)

S2 Dataset Fasta file of all sequences available. (FAS)

## References

[1] Muscatine L. The role of symbiotic algae in carbon and energy flux in reef corals In: Dubinsky Z, editor. Ecosystems of the world. 25. The Nederlands: Elsevier Science Publishing Company; 1990 p. 75–87.

[2] Hughes TP, Kerry JT, Álvarez-Noriega M, Álvarez-Romero JG, Anderson KD, Baird AH, et al Global warming and recurrent mass bleaching of corals. Nature. 2017;543(7645):373–7. 10.1038/nature21707 28300113

[3] Hoegh-Guldberg O. Climate change, coral bleaching and the future of the world’s coral reefs. Marine and Freshwater Research. 1999;50:839–66.

[4] Buddemeier RW, Fautin DG. Coral bleaching as an adaptive mechanism: a testable hypothesis. Bioscience. 1993;43(320–326):320–6.

[5] Berkelmans R, van Oppen MJH. The role of zooxanthellae in the thermal tolerance of corals: a ‘nugget of hope’ for coral reefs in an era of climate change. Proceedings of the Royal Society Biological Sciences 2006;273:2305–12. 10.1098/rspb.2006.3567 16928632 PMC1636081

[6] Silverstein RN, Cunning R, Baker AC. Change in algal symbiont communities after bleaching, not prior heat exposure, increases heat tolerance of reef corals. Global change biology. 2015;21(1):236–49. 10.1111/gcb.12706 25099991

[7] LaJeunesse TC, Parkinson JE, Gabrielson PW, Jeong HJ, Reimer JD, Voolstra CR, et al Systematic Revision of Symbiodiniaceae Highlights the Antiquity and Diversity of Coral Endosymbionts. Current Biology. 2018;28(16):2570–80.e6. 10.1016/j.cub.2018.07.008. 10.1016/j.cub.2018.07.008 30100341

[8] LaJeunesse TC, Pettay DT, Sampayo EM, Phongsuwan N, Brown B, Obura DO, et al Long-standing environmental conditions, geographic isolation and host-symbiont specificity influence the relative ecological dominance and genetic diversification of coral endosymbionts in the genus *Symbiodinium*. Journal of Biogeography. 2010;37(5):785–800. 10.1111/j.1365-2699.201002273.x

[9] Oliver TA, Palumbi SR. Many corals host thermally resistant symbionts in high-temperature habitat. Coral Reefs. 2011;30(1):241–50.

[10] Hsu C-M, Keshavmurthy S, Dennis V, Kuo C-Y, Wang J-T, Meng P-J, et al Temporal and spatial variations of symbiont communities in catch bowl coral, Isopora palifera (Scleractinia; Acroporidae), at reefs in Kenting National Park, Taiwan. Zoological Studies. 2012;51(8):1343–53

[11] Keshavmurthy S, Meng P-J, Wang J-T, Kuo C-Y, Yang S-Y, Hsu C-M, et al Can resistant coral-*Symbiodinium* associations enable coral communities to survive climate change? A study of a site exposed to long-term hot water input. Peer J. 2014;2:e327 10.7717/peerj.327 24765567 PMC3994648

[12] Chen CA, Lam KK, Nakano Y, Tsai W-S. A stable association of the stress-tolerant zooxanthellae, *Symbiodinium* clade D, with the low-temperature-tolerant coral, *Oulastrea crispata* (Scleractinia: Faviidae) in subtropical non-reefal coral communities. Zoological Studies. 2003;42(4):540–50.

[13] Lien Y-T, Nakano Y, Plathong S, Fukami H, Wang J-T, Chen CA. Occurrence of the putatively heat-tolerant *Symbiodinium* phylotype D in high-latitudinal outlying coral communities. Coral Reefs. 2007;26(1):35–44.

[14] Lien Y-T, Keshavmurthy S, Nakano Y, Sakanan P, Huang H, Hsu C-M, et al Host genetics and *Symbiodinium* D diversity in a stress-tolerant scleractinian coral, *Oulastrea crispata*, in the West Pacific. Marine Ecology Progress Series. 2013;473: 163–77.

[15] Baker A, McClanahan T, Starger C, Boonstra R. Long-term monitoring of algal symbiont communities in corals reveals stability is taxon dependent and driven by site-specific thermal regime. Marine Ecology Progress Series. 2013;479:85–97. 10.3354/meps10102

[16] Jones AM, Berkelmans R, van Oppen MJH, Mieog JC, Sinclair W. A community change in the algal endosymbionts of a scleractinian coral following a natural bleaching event: field evidence of acclimatization. Proceedings of the Royal Society Biological Sciences 2008;275:1359–65. 10.1098/rspb.2008.0069 18348962 PMC2367621

[17] Lajeunesse TC, Smith RT, Finney J, Oxenford H. Outbreak and persistence of opportunistic symbiotic dinoflagellates during the 2005 Caribbean mass coral ‘bleaching’ event. Proceedings of the Royal Society Biological Sciences 2009;276(1676):4139–48. 10.1098/rspb.2009.1405 19740874 PMC2821356

[18] Stat M, Loh WKW, LaJeunesse TC, Hoegh-Guldberg O, Carter DA. Stability of coral-endosymbiont associations during and after a thermal stress event in the southern Great Barrier Reef. Coral Reefs. 2009;28(3):709–13. Epub 713

[19] Thornhill DJ, Xiang Y, Fitt WK, Santos SR. Reef endemism, host specificity and temporal stability in populations of symbiotic dinoflagellates from two ecologically dominant Caribbean corals. PLos ONE. 2009;4(7):e6262 10.1371/journal.pone.0006262 19603078 PMC2706050

[20] Fisher PL, Malme MK, Dove S. The effect of temperature stress on coral-Symbiodinium associations containing distinct symbiont types. Coral Reefs. 2012;31(2):473–85.

[21] Levas SJ, Grottoli AG, Hughes A, Osburn CL, Matsui Y. Physiological and biogeochemical traits of bleaching and recovery in the mounding species of coral Porites lobata: Implications for resilience in mounding corals. PloS one. 2013;8(5):e63267 10.1371/journal.pone.0063267 23658817 PMC3642184

[22] Baker AC. Flexibility and specificity in coral-agal symbiosis: diversity, ecology, and biogeogrpahy of *Symbiodinium*. Annual Reviews of Ecology, Evolution, and Systematics. 2003;34:661–89.

[23] Silverstein RN, Correa AMS, Baker AC. Specificity is rarely absolute in coral−algal symbiosis: implications for coral response to climate change. Proceeding of Royal Society Biology. 2012;279:2609–18. 10.1098/rspb.2012.0055 PMC3350700 22367985

[24] Goulet TL. Most corals may not change their symbionts. Marine Ecology Progress Series. 2006;321:1–7.

[25] Cunning R, Silverstein RN, Baker AC. Symbiont shuffling linked to differential photochemical dynamics of Symbiodinium in three Caribbean reef corals. Coral Reefs. 2017:1–8.

[26] Mieog JC, van Oppen MJ, Cantin NE, Stam WT, Olsen JL. Real-time PCR reveals a high incidence of Symbiodinium clade D at low levels in four scleractinian corals across the Great Barrier Reef: implications for symbiont shuffling. Coral Reefs. 2007;26(3):449–57.

[27] Mieog JC, van Oppen MJH, Berkelmans R, Stam WT, Olsen JL. Quantification of algal endosymbionts (*Symbiodinium*) in coral tissue using real-time PCR. Molecular Ecology Resources. 2009;9:74–82. 10.1111/j.1755-0998.2008.02222.x 21564569

[28] Rowan R, Knowlton N. Intraspecific diversity and ecological zonation in coral-algal symbiosis. Procceeding of the National Academy of Sciences of the United States of America. 1995;92:2850–3. 10.1073/pnas.92.7.2850 PMC42316 7708736

[29] Rowan R, Knowlton N, Baker A, Jara J. Landscape ecology of algal symbionts creates variation in episodes of coral bleaching. Nature. 1997;388:265–9. 10.1038/40843 9230434

[30] Toller WW, Rowan R, Knowlton N. Zooxanthellae of the *Montastraea annularis* species complex: patterns of distribution of four taxa of *Symbiodinium* on different reefs and across depths. Biological Bulletin. 2001;201:348–59. 10.2307/1543613 11751247

[31] Kemp DW, Thornhill DJ, Rotjan RD, Iglesias-Prieto R, Fitt WK, Schmidt GW. Spatially distinct and regionally endemic Symbiodinium assemblages in the threatened Caribbean reef-building coral Orbicella faveolata. Coral Reefs. 2015;34(2):535–47.

[32] Chen CA, Wang J-T, Fang L-S, Yang Y-W. Fluctuating algal symbiont communities in *Acropora palifera* (Scleractinia: Acroporidae) in Taiwan. Marine Ecology Progress Series. 2005;295:113–21.

[33] van Oppen MJH, McDonald BJ, Willis B, Miller DJ. The evolutionary history of the coral genus *Acropora* (Scleractinia, Cnidaria) based on a mitochondrial and a nuclear marker: reticulation incomplete lineage sorting, or morphological convergence? Molecular Biology and Evolution. 2001;18(7):1315–29. 10.1093/oxfordjournals.molbev.a003916 11420370

[34] Ulstrup KE, van Oppen MJH. Geographic and habitat partitioning of genetically distinct zooxanthellae (*Symbiodinium*) in *Acropora* corals on the Great Barrier Reef. Molecular Ecology. 2003;12:3477–84. 14629362 10.1046/j.1365-294x.2003.01988.x

[35] Thornhill DJ, Lajeunesse TC, Kemp DW, Fitt WK, Schmidt GW. Multi-year, seasonal genotypic surveys of coral-algal symbioses reveal prevalent stability or post-bleaching reversion. Marine Biology. 2006;148:711–22.

[36] Warner ME, LaJeunesse TC, Robison JD, Thur RM. The ecological distribution and comparative photobiology of symbiotic dinoflagellates from reef corals in Belize: potential implications for coral bleaching. Limnology and Oceanography. 2006;51(4):1887–97.

[37] Ulstrup KE, Hill R, van Oppen MJH, Larkum AWD, Ralph PJ. Seasonal variation in the photo-physiology of homogeneous and heterogeneous *Symbiodinium* consortia in two scleractinian corals. Marine Ecology Progress Series. 2008;361:139–50. 10.3354/meps07360

[38] Kemp DW, Hernandez-Pech X, Iglesias-Prieto R, Fitt WK, Schmidt GW. Community dynamics and physiology of Symbiodinium spp. before, during, and after a coral bleaching event. Limnology and Oceanography. 2014;59(3):788–97.

[39] Fan KL. The thermal effluent problems of three nuclear power plants in Taiwan. Oceanogr Ser 1991;54:309–403.

[40] Peir J-J. Power Uprate Effect on Thermal Effluent of Nuclear Power Plants in Taiwan In: Tsvetkov P, editor. Nuclear Power—Operation, Safety and Environment. Rijeka: InTech; 2011. p. Ch. 13.

[41] Lee H-J, Chao S-Y, Fan K-L, Wang Y-H, Liang N-H. Tidally induced upwelling in a semi-enclosed basin: Nan Wan Bay. Journal of Oceanography. 1997;53:467–80.

[42] Jan S, Chen C-TA. Potential biogeochemical effects from vigorous internal tides generated in Luzon Strait: A case study at the southernmost coast of Taiwan. Journal of Geophysical Research: Oceans. 2009;114(C4):C04021 10.1029/2008JC004887

[43] Keshavmurthy S, Hsu C-M, Kuo C-Y, Meng P-J, Wang J-T, Chen CA. Symbiont communities and host genetic structure of the brain coral *Platygyra verweyi*, at the outlet of a nuclear power plant and adjacent areas. Molecular Ecology. 2012 21(17):4393–407. 10.1111/j.1365-294X.2012.05704.x 22809041

[44] Kao K-W, Keshavmurthy S, Tsao C-H, Wang J-T, Allen C. Repeated and Prolonged Temperature Anomalies Negate Symbiodiniaceae Genera Shuffling in the Coral Platygyra verweyi (Scleractinia; Merulinidae). Zoological Studies. 2018;57(2018). 10.6620/ZS.2018.57-55 PMC6517774 31966295

[45] Johannes RE, Wiebe WJ. Method for determination of coral tissue biomass and composition. Limnology and Oceanography. 1970:822–4.

[46] Cignoni P, Callieri M, Corsini M, Dellepiane M, Ganovelli F, Ranzuglia G, editors. Meshlab: an open-source mesh processing tool. Eurographics Italian Chapter Conference; 2008.

[47] Fitt WK, McFarland FK, Warner ME, Chilcoat GC. Seasonal patterns of tissue biomass and densities of symbiotic dinoflagellates in reef corals and relation to coral bleaching. Limnology and Oceanography. 2000;45(3):677–85.

[48] St Jeffrey, Humphrey G. New spectrophotometric equations for determining chlorophylls a, b, c1 and c2 in higher plants, algae and natural phytoplankton. Biochemie und Physiologie der Pflanzen. 1975;167(2):191–4.

[49] Ferrara GB, Murgia B, Parodi AM, Valisano L, Cerrano C, Palmisano G, et al The assessment of DNA from marine organisms via a modified salting-out protocol. Cellular & molecular biology letters. 2006;11(2):155–60. Epub 2006/07/19. 10.2478/s11658-006-0013-7 . 16847562 PMC6275604

[50] LaJeunesse TC. Diversity and community structure of symbiotic dinoflagellates from Caribbean coral reefs. Marine Biology. 2002;141:387–400.

[51] R-Core-Team. R: A Language and Environment for Statistical Computing. Vienna, Austria2017. p. R Foundation for Statistical Computing.

[52] Bates D, Maechler M, Bolker B. lme4: Linear mixed-effects models using S4 classes. R package version 0.999999–0. Vienna; 2012.

[53] Cooper TF, Berkelmans R, Ulstrup KE, Weeks S, Radford B, Jones AM, et al Environmental factors controlling the distribution of *Symbiodinium* harboured by the coral *Acropora millepora* on the Great Barrier Reef. PLoS ONE. 2011;6(10):e25536 10.1371/journal.pone.0025536 22065989 PMC3204971

[54] Oliver TA, Palumbi SR. Distributions of stress-resistant coral symbionts match environmental patterns at local but not regional scales. Marine Ecology Progress Series. 2009;378:93–103.

[55] McClanahan TR, Starger CJ, Baker AC. Decadal changes in common reef coral populations and their associations with algal symbionts (Symbiodinium spp.). Marine ecology. 2015;36(4):1215–29.

[56] Bay LK, Doyle J, Logan M, Berkelmans R. Recovery from bleaching is mediated by threshold densities of background thermo-tolerant symbiont types in a reef-building coral. Royal Society open science. 2016;3(6):160322 10.1098/rsos.160322 27429786 PMC4929921

[57] Cunning R, Silverstein R, Baker A. Investigating the causes and consequences of symbiont shuffling in a multi-partner reef coral symbiosis under environmental change. Proceedings of the Royal Society of London B: Biological Sciences. 2015;282(1809):20141725. 10.1098/rspb.2014.1725 PMC4590431 26041354

[58] Stat M, Gates RD. Clade D *Symbiodinium* in scleractinian corals: a "nugget" of hope, a selfish opportunist, an ominous sign, or all of the above? Journal of Marine Biology. 2011;2011 Article ID 730715, 9 pages. 10.1155/2011/730715

[59] Oliver TA, Palumbi SR. Do fluctuating temperature environments elevate coral thermal tolerance? Coral Reefs. 2011;30(2):429–40. 10.1007/s00338-011-0721-y

[60] Warner M, Chilcoat G, McFarland F, Fitt W. Seasonal fluctuations in the photosynthetic capacity of photosystem II in symbiotic dinoflagellates in the Caribbean reef-building coral Montastraea. Marine Biology. 2002;141(1):31–8. 10.1007/s00227-002-0807-8

[61] Xu L, Yu K, Li S, Liu G, Tao S, Shi Q, et al Interseasonal and interspecies diversities of Symbiodinium density and effective photochemical efficiency in five dominant reef coral species from Luhuitou fringing reef, northern South China Sea. Coral Reefs. 2017;36(2):477–87.

[62] Silverstein RN, Cunning R, Baker AC. Tenacious D: Symbiodinium in clade D remain in reef corals at both high and low temperature extremes despite impairment. Journal of Experimental Biology. 2017;220(7):1192–6. 28108671 10.1242/jeb.148239

[63] LaJeunesse TC, Smith R, Walther M, Pinzón J, Pettay DT, McGinley M, et al Host-symbiont recombination versus natural selection in the response of coral-dinoflagellate symbioses to environmental disturbance. Proceedings of the Royal Society B-Biological Sciences 2010. 10.1098/rspb.2010.0385 PMC2982020 20444713

[64] Fagoonee I, Wilson HB, Hassell MP, Turner JR. The dynamics of zooxanthellae populations: a long-term study in the field. Science. 1999;283:843–5. 10.1126/science.283.5403.843 9933167

[65] Sawall Y, Al-Sofyani A, Banguera-Hinestroza E, Voolstra CR. Spatio-temporal analyses of Symbiodinium physiology of the coral Pocillopora verrucosa along large-scale nutrient and temperature gradients in the Red Sea. PloS one. 2014;9(8):e103179 10.1371/journal.pone.0103179 25137123 PMC4138093

[66] Brown BE, Dunne RP, Ambarsari I, Tissier MDAL, Satapoomin U. Seasonal fluctuations in environmental factors and variations in symbiotic algae and chlorophyll pigments in four Indo-Pacific coral species. Marine Ecology Progress Series. 1999;191:53–69.

[67] Scheufen T, Iglesias-Prieto R, Enríquez S. Changes in the number of symbionts and Symbiodinium cell pigmentation modulate differentially coral light absorption and photosynthetic performance. Frontiers in Marine Science. 2017;4:309.

