# Supplementary material for: Temporal variation and photochemical efficiency of species in Symbiodinaceae associated with coral Leptoria phrygia (Scleractinia; Merulinidae) exposed to contrasting temperature regimes
Source: PLoS One. 2019 Jun 28;14(6):e0218801. doi: 10.1371/journal.pone.0218801 (PMC6599219; doi:10.1371/journal.pone.0218801)
Supplement: S1 Table — Months in which sampling was performed are in grey. Abbreviations: MMT° = Mean monthly temperature, MMMax T° = Mean monthly maximum temperature, MMMin T° = Mean monthly minimum temperature, ΔT° = maximum temperature-minimum temperature, VS = Variable Site, and SS = Stable Site. (PDF) [file pone.0218801.s001.pdf]

S1 Table. Seawater temperature information at both sites from June 2016 to June 2017.

Year	Month	MM T° in VS	MM T° in SS	MMMax T° in VS	MMMax T° in SS	MMMin T° in VS	MMMin T° in SS	Mean ΔT° in VS	Mean ΔT° in SS
**2016**	6	30.5 ± 1.5	29.1 ± 0.9	32.0 ± 1.1	29.9 ± 0.8	28.8 ± 1.6	28.3 ± 0.9	3.2 ± 1.1	1.6 ± 0.8
**2016**	7	31.0 ± 1.1	29.7 ± 0.6	32.3 ± 0.9	30.4 ± 0.6	29.4 ± 1.2	28.8 ± 0.6	2.9 ± 1.5	1.6 ± 0.8
**2016**	8	30.4 ± 1.1	29.8 ± 0.5	31.5 ± 0.9	30.2 ± 0.6	29.0 ± 0.9	29.3 ± 0.4	2.5 ± 0.8	0.9 ± 0.4
**2016**	9	29.6 ± 1.1	29.1 ± 0.6	30.6 ± 1.0	29.6 ± 0.6	28.5 ± 0.9	28.7 ± 0.6	2.1 ± 0.6	0.9 ± 0.4
**2016**	10	28.8 ± 0.8	28.4 ± 0.4	29.6 ± 0.5	28.8 ± 0.4	27.9 ± 0.9	28.1 ± 0.4	1.7 ± 0.6	0.7 ± 0.4
**2016**	11	27.4 ± 0.8	26.8 ± 0.7	28.1 ± 0.7	27.4 ± 0.8	26.6 ± 0.8	26.3 ± 0.7	1.5 ± 0.5	1.1 ± 0.3
**2016**	12	26.0 ± 1.2	25.2 ± 0.9	26.9 ± 0.8	25.8 ± 0.9	25.0 ± 1.5	24.6 ± 0.9	1.9 ± 0.8	1.2 ± 0.3
**2017**	1	25.4 ± 0.9	23.7 ± 0.7	26.3 ± 0.9	24.3 ± 0.6	24.6 ± 0.9	23.1 ± 0.7	1.8 ± 0.6	1.2 ± 0.4
**2017**	2	25.7 ± 0.7	24.4 ± 0.9	26.4 ± 0.7	25.0 ± 0.8	24.9 ± 0.6	23.8 ± 0.8	1.5 ± 0.6	1.2 ± 0.3
**2017**	3	26.8 ± 0.9	25.2 ± 0.7	27.8 ± 0.9	25.9 ± 0.6	25.8 ± 0.7	24.6 ± 0.6	2.0 ± 0.6	1.3 ± 0.4
**2017**	4	27.0 ± 0.9	26.1 ± 1.0	27.9 ± 1.0	26.8 ± 0.9	26.1 ± 0.8	25.5 ± 0.9	1.8 ± 0.9	1.4 ± 0.4
**2017**	5	28.3 ± 1.2	27.6 ± 0.9	29.5 ± 1.2	28.3 ± 0.8	26.7 ± 1.3	26.9 ± 0.8	2.9 ± 1.5	1.4 ± 0.5
**2017**	6	29.3 ± 2.0	29.3 ± 0.8	30.6 ± 1.8	29.9 ± 0.8	27.8 ± 1.9	28.9 ± 0.7	2.8 ± 0.8	1.0 ± 0.4

Months in which sampling was performed are in grey. Abbreviations: MMT°=Mean monthly temperature, MMMax T°=Mean monthly maximum temperature, MMMin T°=Mean monthly minimum temperature, ΔT°=maximum temperature-minimum temperature, VS=Variable Site, and SS=Stable Site.
