# Supplementary material for: Temporal variation and photochemical efficiency of species in Symbiodinaceae associated with coral Leptoria phrygia (Scleractinia; Merulinidae) exposed to contrasting temperature regimes
Source: PLoS One. 2019 Jun 28;14(6):e0218801. doi: 10.1371/journal.pone.0218801 (PMC6599219; doi:10.1371/journal.pone.0218801)
Supplement: S2 Table — (PDF) [file pone.0218801.s002.pdf]

S2 Table. Percentage of *Durusdinium* spp. at both sites.

Variable Site				
Colony	Sample	August'16	December'16	March'17
COL2	2-1	100	100	100
	2-2	100	100	100
	2-3	100	99	100
	2-4	100	99	99
	2-5	100	100	100
COL3	3-1	100	100	100
	3-2	100	100	100
	3-3	100	100	100
	3-4	100	100	100
	3-5	100		
COL4	4-1	100	100	100
	4-2	100	100	100
	4-3	99	100	100
	4-4	100	97	100
	4-5	99	100	100
COL5	5-1	96	100	100
	5-2	100	100	100
	5-3	97	100	100
	5-4	100	100	100
	5-5	100	100	99
COL6	6-1	99	100	100
	6-2	98	100	99
	6-3	100	100	99
	6-4	100	97	100
	6-5	100	100	100
COL7	7-1	100	100	100
	7-2	100	100	100
	7-3	99	100	100
	7-4		100	100
	7-5		100	100
COL9	9-1	100	100	100
	9-2	100	100	99
	9-3	100	100	100
	9-4	100	100	100
	9-5	100	100	100
COL10	10-1	59	99	100
	10-2	86	93	80
	10-3	16	78	100
	10-4	26	92	39
	10-5	80	72	63
COL11	11-1	98	94	98
	11-2	100	100	100
	11-3	100	100	100
	11-4	100	98	100
	11-5	100	98	100
COL12	12-1	100	99	100
	12-2	100	100	100
	12-3	100	100	100
	12-4	100	100	100
	12-5	100	98	100
COL13	13-1	100	100	100
	13-2	100	98	100
	13-3	100	99	100
	13-4	100	97	100
	13-5	100	100	100
COL15	15-1	100	93	98
	15-2	97	95	100
	15-3	100	99	100
	15-4	97	98	74
	15-5	77	95	88

Stable Site				
Colony	Sample	August'16	December'16	March'17
COL1	1-1	6	4	98
	1-2	34	50	84
	1-3	68	79	62
	1-4	87	47	100
	1-5	88	99	86
COL2	2-1	48	60	64
	2-2	92	87	84
	2-3	98	99	100
	2-4	91	95	98
	2-5	37	100	99
COL3	3-1	100	95	32
	3-2	87	95	40
	3-3	100	74	91
	3-4	99	99	68
	3-5	100	98	100
COL4	4-1	0	0	0
	4-2	0	22	0
	4-3	1	75	3
	4-4	29	8	10
	4-5	30	0	0
COL5	5-1	0	0	0
	5-2	0	0	0
	5-3	1	0	0
	5-4	0	0	0
	5-5	0	3	0
COL6	6-1	0	0	0
	6-2	0	0	0
	6-3	0	0	0
	6-4	0	0	0
	6-5	0	0	0
COL7	7-1		0	0
	7-2		0	0
	7-3		0	0
	7-4		0	0
	7-5		0	1
COL8	8-1	99	100	100
	8-2	100	100	100
	8-3	100	100	100
	8-4	100	100	100
	8-5	100	100	100
COL9	9-1	1	0	0
	9-2	0	0	0
	9-3	0	0	0
	9-4	0	0	0
	9-5	0	0	0
COL10	10-1		0	0
	10-2		0	0
	10-3		0	0
	10-4		0	0
	10-5		0	0
COL11	11-1	0		0
	11-2	0		0
	11-3	0		0
	11-4	0		0
	11-5	0		0
COL12	12-1	0	0	
	12-2	0	0	
	12-3	0	0	
	12-4	0	0	
	12-5	0	0	
COL16	16-1	0	0	0
	16-2	0	6	3
	16-3	0	7	0
	16-4	7	15	79
	16-5	87	92	100
